# Childhood adversity and midlife suicidal ideation

**DOI:** 10.1017/S0033291716002336

**Published:** 2016-10-20

**Authors:** S. A. Stansfeld, C. Clark, M. Smuk, C. Power, T. Davidson, B. Rodgers

**Affiliations:** 1 Centre for Psychiatry, Wolfson Institute of Preventive Medicine, Barts and the London School of Medicine and Dentistry, Queen Mary University of London, London EC1M 6BQ, UK; 2 Population, Policy and Practice, University College London, Institute of Child Health, 30 Guilford Street, London WC1N 1EH, UK; 3 Centre for Gambling Research, School of Sociology, Beryl Rawson Building, The Australian National University, Acton, ACT 2601, Australia; 4 School of Demography, The Australian National University, Acton, ACT 2601, Australia

**Keywords:** Adversity, childhood psychological disorders, cohort studies, suicide

## Abstract

**Background:**

Childhood adversity predicts adolescent suicidal ideation but there are few studies examining whether the risk of childhood adversity extends to suicidal ideation in midlife. We hypothesized that childhood adversity predicts midlife suicidal ideation and this is partially mediated by adolescent internalizing disorders, externalizing disorders and adult exposure to life events and interpersonal difficulties.

**Method:**

At 45 years, 9377 women and men from the UK 1958 British Birth Cohort Study participated in a clinical survey. Childhood adversity was prospectively assessed at the ages of 7, 11 and 16 years. Suicidal ideation at midlife was assessed by the depressive ideas subscale of the Revised Clinical Interview Schedule. Internalizing and externalizing disorders were measured by the Rutter scales at 16 years. Life events, periods of unemployment, partnership separations and alcohol dependence were measured through adulthood.

**Results:**

Illness in the household, paternal absence, institutional care, parental divorce and retrospective reports of parental physical and sexual abuse predicted suicidal ideation at 45 years. Three or more childhood adversities were associated with suicidal ideation at 45 years [odds ratio (OR) 4.31, 95% confidence interval (CI) 2.67–6.94]. Psychological distress at 16 years partially mediated the associations of physical abuse (OR 3.41, 95% CI 2.29–5.75), sexual abuse (OR 4.99, 95% CI 2.90–11.16) with suicidal ideation. Adult life events partially mediated the association of parental divorce (OR 6.34, 95% CI −7.16 to 36.75) and physical (OR 9.59, 95% CI 4.97–27.88) and sexual abuse (OR 6.59, 95% CI 2.40–38.36) with suicidal ideation at 45 years.

**Conclusions:**

Adversity in childhood predicts suicidal ideation in midlife, partially mediated by adolescent internalizing and externalizing disorders, adult life events and interpersonal difficulties. Understanding the pathways from adversity to suicidal ideation can inform suicide prevention and the targeting of preventive interventions.

## Introduction

Adversity in childhood, such as parental divorce and sexual abuse, predicts increased risk of affective disorders, suicidal ideation and completed suicide in adolescence and early adulthood (Fergusson *et al.* 2000; Agerbo *et al.* 2002). Exposure to adversity can interact with genetic factors and result in altered hormonal responses to subsequent stressful life events and may have long-term consequences across the lifecourse (Bradley *et al.* 2008; McGowan *et al.* 2009; Hornung & Heim, 2014). It is unknown whether the association of childhood adversity and suicidal ideation extends into midlife although cross-sectional studies show associations of retrospectively recalled childhood adversity and suicide in adulthood (Dube *et al.* 2001; Bifulco *et al.* 2002; Fanous *et al.* 2004; Corcoran *et al.* 2006; Enns *et al.* 2006; Fairweather *et al.* 2007; Heider *et al.* 2007; Afifi *et al.* 2008) and sexual and physical abuse in childhood (Fanous *et al.* 2004; Ystgaard *et al.* 2004). In this context, retrospective recall of childhood adversity has been found to be biased by contemporaneous adult mental ill-health (Colman *et al.* 2016).

Little is known of potential pathways between childhood adversity and adult suicidal ideation but pathways via adult affective disorders may be relevant. The association of childhood adversity with adult suicidal ideation may be mediated through early onset of psychopathology (Fanous *et al.* 2004). Early-onset psychopathology is associated with increased risk of psychopathology in adulthood (Clark *et al.* 2007) and repeated internalizing disorders in childhood are associated with greatly increased risk of further psychopathology across the lifecourse and hence with suicidal ideation (Colman *et al.* 2007). Alternatively, the association of childhood adversity with adult suicidal ideation may be mediated through increased exposure to life events in adulthood (Fergusson *et al.* 2000), where early adversity exposure predicts adulthood adversity exposure either through trajectories of social disadvantage (Graham & Power, 2004) or through selection of adverse environments (Kendler & Eaves, 1986).

Kendler's developmental model for major depression in women (Kendler *et al.* 2002) and men (Kendler *et al.* 2006) includes three broad pathways: through the development of internalizing disorders, externalizing disorders, and adult interpersonal difficulties (adult adversity). This developmental model tested on twin samples has been innovative, integrating the multifactorial aetiology of adult depression, incorporating both genetic and environmental influences. The model explains a large proportion of the variance in the liability for adult depressive episodes in women (52%) and men (49%). The final model was very similar in women and men. In all, 18 predictor variables were organized across five tiers: childhood (genetic risk, disturbed family environment, sexual abuse, parental loss); early adolescence (neuroticism, self-esteem, anxiety and conduct disorder); late adolescence (educational attainment, lifetime traumas, social support, substance misuse); adulthood (divorce, history of major depression); and last year (marital problems, independent/dependent life events) (Kendler *et al.* 2002). Neuroticism and early-onset anxiety disorders were the most powerful influences in the internalizing pathway, with conduct disorder and substance misuse being most powerful in the externalizing pathway. The adult interpersonal difficulty pathway was more complex: childhood adversity influenced low educational attainment, lifetime trauma and low social support. The authors comment that ‘many of the depressogenic consequences of the earlier adversities appear to be in the realm of troubled interpersonal relationships’ (Kendler *et al.* 2002).

We examined these three pathways for the associations between childhood adversity at 7, 11 and 16 years and suicidal ideation at 45 years in participants from the British National Childhood Development Study (1958 Birth Cohort). We hypothesized that: (1) childhood adversity predicts suicidal ideation at midlife; and (2) the association of childhood adversity and suicidal ideation is partially mediated through (*a*) internalizing disorders in childhood, (*b*) externalizing disorders in childhood and (*c*) increased exposure to life events and interpersonal difficulties in adulthood.

## Method

### Setting

Data were from the 1958 British Birth Cohort, a study of 98% of births in England, Scotland and Wales during 1 week in 1958 (*n* = 17 416). Analyses were based on 9377 participants in a clinical survey at 45 years. The response rate for the clinical survey was 78% of those invited, representing 54% of the surviving population (Power & Elliott, 2006). Information was available from parents and schools at ages 7, 11 and 16 years and by participant interviews at ages 7, 11, 16, 23, 33 and 42 years. Ethical approval for the survey was given by the South East Multi-Centre Research Ethics Committee: informed consent was obtained. The authors assert that all procedures contributing to this work comply with the ethical standards of the relevant national and international committees on human experimentation and with the Helsinki Declaration of 1975, as revised in 2008.

### Measures

#### Midlife suicidal ideation

Two measures captured suicidal ideation in midlife: depressive ideas and a single-item measure on whether life is worth living. Depressive ideas were assessed using the Revised Clinical Interview Schedule (CIS-R) (Lewis *et al.* 1992), administered by a trained research nurse using computer-assisted personal interviewing in the home-based clinical examination at 45 years. The CIS-R measures International Classification of Diseases, tenth edition (ICD-10) affective and anxiety diagnoses in the past week. Two measures of suicidal ideation were derived from CIS-R data. A ‘depressive ideas’ scale in the past 7 days was formed by summing affirmative answers to the following questions: ‘Have you on at least one occasion felt guilty or blamed yourself when it hasn't been your fault?’; ‘During the past week have you been feeling you are not as good as other people?’; ‘During the past week have you felt hopeless about your future?’; ‘In the past week have you felt that life isn't worth living?’; In the past week have you thought of killing yourself?’. This five-point scale is dichotomized at ⩾2 to indicate a clinically significant level of depressive ideas based on CIS-R scoring protocols (Lewis *et al.* 1992). A single item from this scale was also examined as an outcome: ‘In the past week have you felt that life is not worth living?’ – analysed as a dichotomous outcome.

#### Childhood adversity

Childhood adversity was defined as exposure to traumatic events or chronic stressors (Tiet *et al.* 1998). Prospectively assessed measures of childhood adversity were available from data collected at 7, 11 and 16 years from the cohort member, their parent (usually the mother), the interviewer, the school/teacher and medical examination. These items are detailed elsewhere (Clark *et al*. 2010), but in brief:

Illness relates to any mental or physical illness within the child's household at 7, 11 or 16 years;
Neglected appearance: medical report of the child having an underfed/neglected appearance at 7 and/or 11 years;
Maternal absence: any report of the child not living with their natural mother at 7, 11 and/or 16 years, through death, divorce, separation, illegitimacy or being in institutional care;
Paternal absence similarly reported the father's absence;
In care: the child having been in care at 7, 11 and/or 16 years;
Parental divorce: at 33 years participants reported parental divorce by 16 years;
Physical abuse by a parent (‘I was physically abused by a parent – punched, kicked, hit, or beaten with an object or needed medical treatment’);
Sexual abuse by a parent (‘I was sexually abused by a parent’).

Physical abuse and sexual abuse were ascertained from retrospective report at 45 years (Rosenman & Rodgers, 2004) as these data were not collected prospectively. These types of adversity are difficult to assess prospectively because children often do not report abuse at the time because of shame and adult coercion. A measure of cumulative adversity counted the reports of neglected appearance, in care, parental divorce, parental physical abuse, parental sexual abuse, and maternal and paternal absence (0, 1, 2, 3 or more): the latter two adversities were only included if parental divorce and being in care were not reported (see Clark *et al*. 2010). This cumulative measure attempts to account for exposure to multiple adversities across childhood.

#### Childhood internalizing and externalizing disorders

Internalizing and externalizing problems at 16 years were measured using the teacher version of the Rutter scales (Rutter, 1967; Elander & Rutter, 1996). Two scales were formed by summing and square root transforming the items ‘worries’, ‘solitary’, ‘miserable’, ‘fearful’ and ‘fussy’ for internalizing problems and ‘destructive’, ‘fights’, ‘not much liked by other children’, irritable’, ‘disobedient’, ‘lies’, ‘steals’, ‘resentful/aggressive’ and ‘bullies’ for externalizing problems. A score in the top 13% was defined as internalizing and externalizing disorders, the lowest 50% were not a case, and the remainder were borderline based on earlier studies (Ghodsian, 1983; Clark *et al.* 2007). These scales have demonstrated reliability in this cohort (Cronbach's *α* for internalizing problems scale = 0.66, *n* = 7225; Cronbach's *α* for externalizing problems scale = 0.88, *n* = 7179) (Clark *et al.* 2007).

### Adult stressors and interpersonal difficulties

#### Recent life events

At 45 years 13 questions from the Australian Personality and Total Health (PATH) Study (Windsor *et al.* 2008) measured serious illnesses and death within the family, relationship problems, work problems, unemployment, financial problems and legal/police problems. Responses were ‘yes’ or ‘no’. A sum of the number of life events in the past 6 months was coded as 0 or ⩾1. More detailed classification of life events in the last 6 months was not possible because of the small numbers of events. Social support was assessed by a question at 42 years where participants indicated whether they had someone they could turn to for advice/support (yes/no).

#### Lifecourse unemployment

A count of times the participant had been unemployed (having no employment as opposed to undertaking other non-working activities such as homemaking) from 16 to 42 years was calculated from the activity histories available from 1974 to 2009 (Hancock *et al.* 2011*a*). This variable was coded as 0, 1, 2 or 3 or more periods of unemployment.

#### Lifecourse partnerships

A count of the number of separations from a cohabiting partnership from 16 to 42 years was derived from the partnership histories for the period 1974–2008 available for the cohort (Hancock *et al.* 2011*b*). This variable was coded as 0 or 1 *v.* 2 or more separations.

#### Problem drinking

Problem drinking was included as an indicator of adult externalizing behaviours. A dichotomous variable was derived indicating problem drinking in the past year at 33 or 42 years as indicated by affirmative responses to two or more of the CAGE items (Cutting down, Annoyance by criticism, Guilty feeling, and Eye-openers) (Mayfield *et al.* 1974): ‘They felt they should cut down’; ‘People annoyed them by criticizing their drinking’; ‘They felt bad or guilty about their drinking’; and ‘They drank in the morning to get rid of a hangover’.

#### Long-standing illness

At 42 years participants indicated if they had ‘any long-standing illness, disability or infirmity’, coded as yes *v.* no.

### Socio-economic position and educational qualifications

Adult social position was based on current/most recent occupation at 42 years and categorized using the British Registrar General classification (Office of Population Censuses and Surveys, 1980) as: ‘I and II’, professional/managerial/technical; ‘IIINM’, other non-manual; ‘IIIM’, skilled manual; and ‘IV and V’, unskilled manual. The same measure was available for the participant's father at birth: children with an absent father at birth were coded as ‘IV and V’. This classification was also available for the participant's father at birth (Stansfeld *et al.* 2008). Qualifications were reported at 33 years, categorized as none, ‘O’ levels, and ‘A’ levels or higher.

### Statistical analysis

Analyses were carried out using STATA (version 13; StataCorp LP, USA).

#### Imputation

Multiple imputation, under the ‘missing at random’ assumption, was used to address the issue of missing data, using the ICE program in STATA. The measures described above were included in the imputation equations. Employment status at 45 years, and social class at 7 and 42 years were also included as they were significantly associated with attrition (Atherton *et al.* 2008). Missing data on the variables ranged from 0 to 20%, except for externalizing disorders at 16 years (22.9%), illness in the family (26.2%), in care (38.9%) and neglect (56.1%) (Table 1). All participants except 1245 who had died by 45 years were included in the imputation, but analyses in this paper are restricted to participants in the 45-year study (*n* = 9377). A total of 30 datasets of the imputation were run and analyses indicated that the measures were stable across the imputations. Parameter estimates from the 30 imputations were estimated using the MIM function in STATA. Imputed analyses are presented with unimputed prevalence figures.

**Table 1.** Unimputed frequencies and percentages for the key variables in the analyses

Variable	No. with characteristic for biomedical sample at 45 years (*n* = 9377)	Percentage with characteristic for biomedical sample at 45 years (*n* = 9377)^a^	Percentage with missing data out of *n* = 9377
Depressive ideas scale ⩾2	675	7.2	0.9
Thought that life isn't worth living in the past week	189	2.0	0.9
Illness in the household (7–16 years)	1415	15.1	26.2
Neglected/underfed appearance (7–11 years)	461	4.9	56.1
Maternal absence (7–16 years)	435	4.6	23.1
Paternal absence (7–16 years)	1125	12.0	21.8
In care (7–16 years)	307	3.3	38.9
Divorce of parents by age 16 years	882	9.4	11.2
Parental physical abuse^b^	563	6.0	0.7
Parental sexual abuse^b^	149	1.6	0.7
Life events at 42 years			2.5
0	4480	47.8	
1 or more	4601	49.1	
No of cohabiting partnership separations			2.6
0 or 1	8936	95.3	
2 or more	441	4.7	
No of periods of unemployment			0.0
0	5911	63.0	
1	1981	21.1	
2	672	7.2	
3	575	6.1	
CAGE – problems in past year at 33 years (score of 2 or more in either year)	570	6.0	11.3
CAGE – problems in past year at 42 years (score of 2 or more in either year)	720	7.6	4.5
Emotional support at 42 years (no)	298	3.2	3.1
Any long-standing illness at 42 years	2653	28.3	3.1
Gender (female)	4715	50.3	0.0
Social class at 42 years			16.7
I and II	3453	36.8	
IIInm	1657	17.7	
IIIm	1539	16.4	
IV and V	1161	12.4	
Qualifications at 33 years			12.9
None	632	6.7	
O levels	3654	39.0	
A levels or higher	3872	41.3	
Externalizing at 16 years	803	8.5	22.9
Internalizing at 16 years	1092	11.6	16.6
Externalizing or internalizing at 16 years	1594	17.0	22.4

CAGE, Cutting down, Annoyance by criticism, Guilty feeling, and Eye-openers; nm, non-manual; m, manual.

a The percentages are given out of 9377 for each variable: this will underestimate the prevalence for variables with large amounts of missing data, as missing data are included in the total percentage out of 9377.

b Retrospectively reported at 45 years.

#### Modelling

Initial logistic regression analyses, adjusting for gender, qualifications at 33 years and social position at 42 years, examined the strength of the associations between each childhood adversity measure and suicidal ideation at 45 years.

A series of logistic regression analyses were then run to test the hypothesized pathways between each of the childhood adversity measures and suicidal ideation. First, to establish whether the associations of the childhood adversity with suicidal ideation were partially mediated through internalizing and externalizing disorders, we conducted logistic regression analyses of the associations of the childhood adversities on internalizing and externalizing disorders at 16 years adjusted for gender and social class at birth. We conducted logistic regression analyses to establish whether the association of childhood adversity with suicidal ideation is through increased exposure to life events. In order to do this we examined the associations of each childhood adversity measure with recent life events at 45 years, adjusting for gender, qualifications at 33 years, and social position at 42 years.

Further logistic regression analyses assessed the associations of: (i) interpersonal difficulties (the number of partnerships, number of periods unemployed, lack of emotional support at 42 years, long-standing illness at 42 years); and (ii) adult drinking problems (at 33 or 42 years) with midlife suicidal ideation, adjusting for gender, social class at 42 years and qualifications at 33 years. Finally, the associations between childhood adversities and suicidal ideation were re-run, additionally adjusting for (*a*) internalizing and externalizing disorders at 16 years and (*b*) the adulthood life events and interpersonal difficulties.

Estimated causal mediation of the associations between each childhood adversity measure and midlife suicidal ideation by: (i) internalizing disorders; (ii) externalizing disorders; or (iii) interpersonal difficulties and problem drinking was investigated using the *medeff* package (Hicks & Tingley, 2011). We applied 200 bootstrapping replications, to each imputed dataset separately using 2000 simulations in each to approximate the quasi-Bayesian uncertainty parameter estimate; causal mediation estimates were then combined using Rubin's rules (Rubin, 1987). At present mediation can only be examined easily for binary and continuous variables, so mediation analyses are not presented for the multinomial cumulative childhood adversity measure.

All analyses tested for interactions between childhood adversity and gender: analyses were stratified by gender if the interaction was significant (*p* < 0.05) and adjusted for gender where not significant (*p* ⩾ 0.05).

## Results

### Descriptives

Table 1 shows the unimputed frequencies for the key variables. At 45 years, 2.0% had felt that life was not worth living in the past week and 7.2% scored 2 or more on the depressive ideas scale.

### Childhood adversities association with suicidal ideation

Table 2 shows the odds ratios (ORs) for suicidal ideation at 45 years, for each childhood adversity adjusted for gender, qualifications at 33 years and social class at 42 years. All the childhood adversities, except maternal absence, were significantly associated with increased odds of depressive ideas. Physical abuse by a parent and sexual abuse by a parent were associated with a 3-fold increased odds of reporting depressive ideas. All of the childhood adversities were significantly associated with increased odds of feeling life is not worth living in the past week, except maternal absence and neglected appearance. There was an interaction by gender for the effect of divorce of parents on having felt life not worth living in the past week, with an association for males [adjusted OR (AOR) 2.94, 95% confidence interval (CI) 1.68–5.13] but not females. The odds of suicidal ideation increased with the number of childhood adversities reported.

**Table 2.** Increase in risk for (a) suicidal ideation and depressive ideas at 45 years, (b) recent life events and (c) psychological ill health at 16 years, for the presence of each individual adversity/maltreatment^a^

	Suicidal ideation		Recent life events	Psychological ill health at 16 years		
Adversity	In the past week have you felt that life isn't worth living?	Depressive ideas	Life events at 45 years, 1 *v.* 0	Internalizing or externalizing at 16 years	Internalizing at 16 years	Externalizing at 16 years
Illness in the household	2.23 (1.59–3.14)***	1.38 (1.11–1.70)**	1.12 (1.00–1.26)*	1.53 (1.33–1.75)***	1.46 (1.26–1.69)***	1.58 (1.30–1.91)***
Neglected/underfed appearance at 7–11 years	1.61 (0.88–2.95)	1.47 (1.00–2.14)*	1.05 (0.83–1.33)	2.76 (2.22–3.42)***	2.36 (1.85–3.00)***	2.73 (2.03–3.67)***
Maternal absence	0.97 (0.51–1.83)	1.02 (0.72–1.43)	1.02 (0.85–1.23)	1.61 (1.29–2.01)***	1.58 (1.19–2.08)**	1.68 (1.27–2.22)***
Paternal absence	2.12 (1.49–3.00)***	1.34 (1.08–1.66)**	1.11 (0.98–1.26)	1.66 (1.43–1.93)***	1.50 (1.26–1.79)***	1.91 (1.59–2.30)***
In care	2.17 (1.26–3.74)**	1.60 (1.11–2.31)*	1.42 (1.14–1.77)**	2.54 (2.03–3.17)***	2.22 (1.73–2.86)***	2.87 (2.20–3.76)***
Divorce of parents by age 16 years^b^	1.82 (1.24–2.66)**†	1.32 (1.04–1.68)*	1.19 (1.03–1.38)*	1.75 (1.48–2.08)***	1.53 (1.26–1.87)***	2.09 (1.69–2.57)***
Males	2.94 (1.68–5.13)***					
Females	1.29 (0.75–2.22)					
Parental physical abuse^c^	3.07 (2.07–4.56)***	2.83 (2.23–3.59)***	1.78 (1.49–2.14)***	1.77 (1.44–2.19)***	1.30 (1.01–1.68)*	2.42 (1.91–3.07)***
Parental sexual abuse^c^	3.55 (1.95–6.46)***	3.06 (2.05–4.54)***	1.64 (1.16–2.32)**	2.75 (1.88–4.02)***	2.10 (1.38–3.20)***	3.77 (2.50–5.69)***
Cumulative adversity scale at 7–16 years^d^						
1	1.84 (1.13–2.98)*	1.43 (1.10–1.85)**	1.15 (1.02–1.30)*	1.50 (1.28–1.74)***	1.38 (1.16–1.63)***	1.64 (1.30–2.07)***
Males			1.14 (0.95–1.38)			
Females			1.16 (0.99–1.35)			
2	2.56 (1.53–4.29)***	1.56 (1.17–2.08)**	1.24 (1.05–1.46)*	1.95 (1.57–2.42)***	1.63 (1.28–2.09)***	2.34 (1.76–3.12)***
Males			1.17 (0.92–1.47)			
Females			1.31 (1.03–1.66)*			
3 or more	4.31 (2.67–6.94)***	2.24 (1.71–2.93)***	1.39 (1.17–1.66)***††	3.25 (2.64–4.00)***	2.60 (2.06–3.28)***	4.09 (3.21–5.23)***
Males			1.07 (0.83–1.39)			
Females			1.80 (1.42–2.28)***			

Data are given as adjusted odds ratio (95% confidence interval).

a Suicidal ideation and recent life events models adjusted for social class at 42 years, qualifications at 33 years, and gender. Psychological ill health at 16 years model adjusted for gender and social class at 7 years.

b Reported at 33 years.

c Reported at 45 years.

d Measure of adversity only counts absent mother and absent father if being in care and divorce are not present; also does not include illness as this is too frequent. All measures are prospective (measured in childhood) except divorce of parents by age 16 years, parental physical abuse and parental sexual abuse. In all analyses the reference group is no exposure to the adversity or 0 for the cumulative adversity measure.

* *p* ⩽ 0.05, ** *p* ⩽ 0.01, *** *p* ⩽ 0.001.

No significant gender interactions were observed with the following exceptions: † *p* = 0.032; †† *p* = 0.004.

### Childhood adversities and recent life events at 45 years

Table 2 shows the AORs of reporting any recent life event at 45 years for each childhood adversity measure. Associations were observed between illness in the household, being in care, divorce by age 16 years, physical abuse and sexual abuse on recent life events at 45 years. The cumulative number of childhood adversities reported was only associated with recent life events for females, with two adversities or three or more adversities significantly increasing the odds for recent life events (AOR 1.31, 95% CI 1.03–1.66; AOR 1.80 95% CI 1.42–2.28, respectively).

### Childhood adversities and psychological distress at 16 years

After adjustment for gender and social class at birth, all the childhood adversities were associated with externalizing and internalizing problems at 16 years (Table 2). As associations were similar for the two measures at 16 years, further analyses combined ‘any externalizing or internalizing problems at 16 years’ (yes/no). All childhood adversities were associated with psychological distress at 16 years, the strength of the association increasing as the number of adversities increased.

### Adulthood stressors and midlife suicidal ideation

Table 3 shows that all the adulthood stressors were significantly associated with suicidal ideation at 45 years; cohabiting partnership separations, periods of unemployment, problem drinking and long-standing illness increased the odds of depressive ideas around 1.5- to 2-fold. Low emotional support only increased the odds of depressive ideas for females.

**Table 3.** Increase in odds for suicidal ideation and depressive ideas at 45 years, for no. of partnerships, no. of periods unemployed, drinking problems at 33 or 42 years, lack of emotional support at 42 years, long-standing illness at 42 years, internalizing problems and externalizing problems at 16 years^a^

		Suicidal ideation	
Adversity: adult stressor indicator		In the past week have you felt that life isn't worth living?	Depressive ideas
No. of cohabiting partnership separations, 16–42 years	2+	1.96 (1.18–3.24)**	1.74 (1.29–2.37)***
No. of periods of unemployment, 16–42 years	1	1.91 (1.37–2.68)***	1.39 (1.14–1.68)***
	Males	1.58 (0.86–2.90)	
	Females	2.14 (1.43–3.20)***	
	2	1.45 (0.83–2.54)	1.51 (1.13–2.01)**
	Males	1.73 (0.78–3.87)	
	Females	1.28 (0.58–2.84)	
	3+	1.95 (1.15–3.32)*†^b^	1.79 (1.32–2.42)***
	Males	2.96 (1.55–5.63)***	
	Females	0.55 (0.13–2.27)	
CAGE – problems in past year either at 33 or 42 years (score of 2 or more in either year)	2+	2.68 (1.87–3.83)***	2.25 (1.83–2.77)***
Emotional support at 42 years	No	1.48 (0.76–2.89)	1.89 (1.31–2.73)***†^c^
	Males		1.39 (0.81–2.38)
	Females		2.90 (1.70–4.93)***
Any long-standing illness at 42 years	Yes	2.44 (1.80–3.30)***	2.07 (1.76–2.44)***
Childhood psychological distress			
Internalizing or externalizing at 16 years	Yes	1.59 (1.08–2.34)*	1.59 (1.30–1.95)***
Internalizing at 16 years	Yes	1.54 (0.99–2.39)	1.61 (1.29–2.02)***
Externalizing at 16 years	Yes	1.63 (1.09–2.43)*	1.58 (1.23–2.04)***†^d^
	Males		1.15 (0.76–1.75)
	Females		1.98 (1.45–2.71)***

Data are given as adjusted odds ratio (95% confidence interval).

CAGE, Cutting down, Annoyance by criticism, Guilty feeling, and Eye-openers.

a Adult stressors model adjusted for gender, social class at 42 years, and qualifications at 33 years. Psychological ill health at 16 years model adjusted for gender and social class at 7 years.

* *p* ⩽ 0.05, **p ⩽ 0.01, ***p ⩽ 0.001.

†^b^ Gender interaction *p* = 0.042, †^c^ gender interaction *p* = 0.034, †^d^ gender interaction *p* = 0.018.

### Internalizing and externalizing disorders as mediating factors

Table 4 shows that after adjustment for any internalizing or externalizing disorder at 16 years and the adulthood stressors, illness in the household, paternal absence, divorce by 16 years, physical abuse, sexual abuse and the cumulative number of adversities remained significantly associated with suicidal ideation. Psychological distress at 16 years mediated these associations by 1.80 to 5.57%. Adulthood problem drinking mediated these associations by 1.75 to 9.22%. Table 4 also shows that after adjustment for the adulthood stressors and psychological distress at 16 years, physical abuse, sexual abuse and the cumulative number of adversities remained significantly associated with depressive ideas at 45 years. Psychological distress at 16 years mediated these associations by 3.41% for physical abuse and 4.99% for sexual abuse. Problem drinking mediated these associations by 2.80% for physical abuse and 6.37% for sexual abuse.

**Table 4.** Adjusted odds ratios (AORs) and 95% confidence intervals (CIs) with supporting mediation effect showing the increase in odds for suicidal ideation and depressive ideas at 45 years, for each increase in the number of reports of the adversities^a^

	In the past week have you felt that life isn't worth living?			Depressive ideas		
Adversity	AOR (95% CI)	Indirect percentage of total mediation effect		AOR (95% CI)	Indirect percentage of total mediation effect	
		Psychological ill health at 16 years	Any drinking problems at 33 or 42 years		Psychological ill health at 16 years	Any drinking problems at 33 or 42 years
		Mean % (95% CI)	Mean % (95% CI)		Mean % (95% CI)	Mean % (95% CI)
Illness in the household	2.00 (1.41–2.83)***	1.80 (1.19–3.22)	1.75 (1.09–3.18)	1.24 (1.00–1.55)		
Neglected/underfed appearance, 7–11 years	1.36 (0.73–2.54)			1.25 (0.83–1.88)		
Maternal absence	0.85 (0.45–1.63)			0.90 (0.63–1.28)		
Paternal absence	1.92 (1.34–2.75)***	2.93 (1.77–6.23)	2.64 (1.58–5.47)	1.19 (0.95–1.49)		
In care	1.74 (0.98–3.11)			1.28 (0.87–1.89)		
Divorce of parents by age 16 years^b^	1.58 (1.07–2.35)*	5.20 (−1.34 to 29.15)	7.18 (−1.86 to 38.99)	1.14 (0.89–1.46)		
Parental physical abuse^c^	2.25 (1.49–3.41)***	3.79 (1.93–11.07)	4.19 (2.12–12.48)	2.13 (1.66–2.74)***	3.41 (2.29–5.75)	2.80 (1.88–4.75)
Parental sexual abuse^c^	2.64 (1.42–4.93)**	5.57 (1.26–32.12)	9.22 (2.57–51.23)	2.32 (1.53–3.53)***	4.99 (2.90–11.16)	6.37 (3.70–14.22)
Cumulative adversity scale						
1	1.67 (1.03–2.70)*			1.30 (1.00–1.69)*		
2	2.21 (1.31–3.74)**			1.34 (1.00–1.80)		
3 or more	3.25 (1.97–5.37)***			1.68 (1.27–2.24)***		

a Adjusted for gender, social class at 42 years, qualifications at 33 years, life events at 45 years, number of partnerships in adulthood, number of periods of unemployment in adulthood, emotional support at 42 years, problem drinking at either 33 or 42 years, and long-standing illness at 42 years and psychological distress at 16 years (any internalizing or externalizing at 16 years).

b Reported at 33 years.

c Reported at 45 years.

* *p* ⩽ 0.05, ** *p* ⩽ 0.01, *** *p* ⩽ 0.001.

### Adult life events and interpersonal difficulties as mediating factors

Table 5 shows the indirect percentage of the total mediation effect of the associations by life events: the associations of childhood adversities with depressive ideas were mediated by 5.59% for illness in the household, 11.79% for physical abuse, and 8.16% for sexual abuse. Cohabiting partnership separations mediated these associations by 2.97% for physical abuse and 2.73% for sexual abuse: the association between illness in the household and depressive ideas was suppressed by cohabiting partnership separations, but only marginally (−0.88%). The total percentage of mediation of the associations with feeling that life is not worth living by life events ranged from 1.55% for illness in the household to 9.59% for physical abuse. The indirect percentage of the mediation effect of these associations by cohabiting partnership separations ranged from 1.27 for paternal absence to 5.22% for physical abuse. Again, the association between illness in the household and suicidal ideation was marginally suppressed by cohabiting partnership separations (−0.50%).

**Table 5.** Adjusted odds ratios (AORs) and 95% confidence intervals (CIs) with supporting mediation effect showing the increase in odds for suicidal ideation and depressive ideas at 45 years, for each childhood adversity^a^

	In the past week have you felt that life isn't worth living?			Depressive ideas		
Adversity	AOR (95% CI)	Indirect percentage of total mediation effect		AOR (95% CI)	Indirect percentage of total mediation effect	
		Life events	Number of partnerships in adulthood		Life events	Number of partnerships in adulthood
		Mean % (95% CI)	Mean % (95% CI)		Mean % (95% CI)	Mean % (95% CI)
Illness in the household	2.00 (1.41–2.83)***	1.55 (1.01–2.78)	−0.50 (−1.11 to −0.17)	1.24 (1.00–1.55)	5.59 (−1.15 to 24.44)	−0.88 (−6.14 to 2.95)
Neglected/underfed appearance at 7–11 years	1.36 (0.73–2.54)			1.25 (0.83–1.88)		
Maternal absence	0.85 (0.45–1.63)			0.90 (0.63–1.28)		
Paternal absence	1.92 (1.34–2.75)***	2.49 (1.45–5.62)	1.27 (0.76–2.91)	1.19 (0.95–1.49)		
In care	1.74 (0.98–3.11)			1.28 (0.87–1.89)		
Divorce of parents by age 16 years^b^	1.58 (1.07–2.35)*	6.34 (−7.16 to 36.75)	2.27 (−3.72 to 14.19)	1.14 (0.89–1.46)		
Parental physical abuse^c^	2.25 (1.49–3.41)***	9.59 (4.97–27.88)	5.22 (2.65–16.38)	2.13 (1.66–2.74)***	11.79 (8.14–19.09)	2.97 (2.01–5.02)
Parental sexual abuse^c^	2.64 (1.42–4.93)**	6.59 (2.40–38.36)	4.71 (0.93–28.69)	2.32 (1.53–3.53)***	8.16 (4.72–18.53)	2.73 (1.57–6.50)
Cumulative adversity scale						
1	1.67 (1.03–2.70)*			1.30 (1.00–1.69)*		
2	2.21 (1.31–3.74)**			1.34 (1.00–1.80)		
3 or more	3.25 (1.97–5.37)***			1.68 (1.27–2.24)***		

a Adjusted for gender, social class at 42 years, qualifications at 33 years, life events at 45 years, number of partnerships in adulthood, number of periods of unemployment in adulthood, emotional support at 42 years, problem drinking at 33 or 42 years, long-standing illness at 42 years and psychological distress at 16 years (any internalizing or externalizing at 16 years).

b Reported at 33 years.

c Reported at 45 years.

* *p* ⩽ 0.05, ** *p* ⩽ 0.01, *** *p* ⩽ 0.001.

We created a combined score describing overall adversity in adulthood. The sum of the dichotomous adversity measures included: number of partnerships, number of periods unemployed, drinking problems at 33 or 42 years, lack of emotional support at 42 years and long-standing illness at 42 years. We adjusted analyses for gender, social class at 42 years and qualifications at 33 years (AOR for life not worth living: cumulative adversity, one adversity AOR 1.75, 95% CI 1.08–2.84; two adversities AOR 2.39, 95% CI 1.42–4.02; three or more adversities AOR 3.79, 95% CI 2.33–6.15; AOR for depressive thoughts: one adversity AOR 1.36, 95% CI 1.05–1.76; two adversities AOR 1.44, 95% CI 1.08–1.92; three or more adversities AOR 1.94, 95% CI 1.47–2.56).

We carried out analyses to examine how much this adult adversity score might mediate the association between childhood adversity and suicidal ideation. The indirect percentage of the total mediation effect was between 3.28 and 8.63 for feeling that life was not worth living and between 7.86 and 8.67 for depressive ideas. The percentage of mediation explained was of little greater magnitude than the individual life events and interpersonal difficulties (see Supplementary Table).

## Discussion

The aims of our study were to assess whether the effects of childhood adversity on suicidal ideation extended beyond adolescence to midlife and whether this was partially mediated by adolescent internalizing disorders, externalizing disorders and adult exposure to life events and interpersonal difficulties. We confirmed that specific childhood adversities which included illness in the household, paternal absence and divorce prospectively predict suicidal ideation at 45 years even after adjustment for confounding and mediating factors. Retrospectively recalled parental sexual and physical abuse also show strong associations with suicidal ideation at 45 years. Childhood adversity predicts adult life events, supporting continuity of exposure to adversity across the lifecourse. Adulthood interpersonal difficulties predicted suicidal ideation.

In terms of investigation of mediation, psychological ill-health in childhood partially mediated the association of illness in the household, paternal absence and physical and sexual abuse. Adult life events partially mediated the association of paternal absence, divorce and physical and sexual abuse, while partnerships in adulthood and problem drinking partially mediated the association of sexual and physical abuse. We have confirmed our hypotheses that some childhood adversities are associated with midlife suicidal ideation and that this is partly mediated by subsequent life events, internalizing and externalizing disorders, although the variance explained in mediation is small. Our findings support Kendler's aetiological framework for affective disorders (Kendler *et al.* 2002, 2006).

Studies of youth suicide have demonstrated the importance of parent-related events in the family such as parental hospital admission with mental illness, parental divorce and marital disruption (Fergusson *et al.* 2000) as risk factors. We found that these risks persist into adulthood in our study, as in other studies (Agerbo *et al.* 2002). The predictive power of parental illness in the household may partially represent genetic transmission of mental illness susceptibility from parent to child as well as the environmental influence of having a sick parent on the child. Early onset of psychopathology, possibly brought forward by adversity, may develop into clinical psychiatric disorder (Clark *et al.* 2007) that persists into adult life perpetuating suicidal risk (Fergusson *et al.* 2000; Dube *et al.* 2001; Fanous *et al.* 2004). Adolescent internalizing disorder and adult suicidal ideation have also been linked in another British cohort study (Colman *et al.* 2007) and adolescent depression and adult psychopathology have been found to be mediating factors for childhood adversity and adult suicidal ideation in the follow-up of the Isle of Wight Study (Pickles *et al.* 2010). Anxiousness and disruptiveness are also mediating factors for childhood adversity and suicidal attempts in young adults (Wanner *et al.* 2012). Altogether, lifecourse persistent psychopathology beginning in adolescence seems a key pathway for midlife suicidal ideation.

In terms of other types of childhood adversity sexual (Fergusson *et al.* 2000; Fanous *et al.* 2004; Ystgaard *et al.* 2004; Afifi *et al.* 2008) and physical abuse (Ystgaard *et al.* 2004; Enns *et al.* 2006; Bruwer *et al.* 2014; Harford *et al.* 2014) have been associated with suicidal ideation, attempts and completed suicide (Séguin *et al.* 2007). Life events in childhood (Fergusson *et al.* 2000) and adulthood, including divorce in adulthood (Dennis *et al.* 2007), are associated with increased risk of suicidal ideation. Interpersonal difficulties in adolescence predict suicidal attempts in young adulthood (Johnson *et al.* 2002). Exposure to childhood adversity is related to increased exposure to midlife events. And, as in our study, there is evidence that self-reported alcohol dependence, an example of externalizing behaviour, partially mediates the effect of sexual and physical abuse (Dube *et al.* 2001).

Lack of maternal and paternal care has been linked to adult suicidal ideation (Enns *et al.* 2006; Heider *et al.* 2007), although we found only an unexpected association with paternal absence, as other studies have found associations with maternal absence, although maternal absence was rare in our population. In this instance the CIs around the estimate will be wide because of the rarity of maternal absence rather than the nature of the effect itself. Despite adjustment for social class and educational attainment some of the effects of paternal absence and divorce on midlife suicidal ideation might relate to financial disadvantage as a consequence of these events in childhood. Bifulco *et al.* (2002) state that all types of childhood adversity increase the risk of adult affective disorder and suicidal ideation noting dose–response relationships and the importance of psychological abuse. In this context the number of adversities experienced may be important for future risk of suicidal ideation (Enns *et al.* 2006), although sexual and physical abuse, as severe adversities, carry greater risk.

The magnitude of the effects of the mediating factors of internalizing, externalizing and interpersonal disorders may have been small due to methodological limitations: risk factor exposure misclassification, lack of key variables at different stages of the lifecourse and lack of coverage of all relevant risk factors within the three groups of mediators. Additionally we needed to dichotomize the mediating variables, rather than use continuous scores because of the skewed nature of their distribution. Thus we may have underestimated the effect of the mediators. Moreover, as the models are fully adjusted we only expected to see small levels of mediation as the other covariates in the model also contribute to the outcome. Looking at individual possible mediators on top of the effects of other covariates means that individual mediators are unlikely to explain a large percentage of the associations. However, in our study we may also be missing some key variables that are shaped by childhood adversity and transmit the risk of suicidal ideation across the lifecourse. One of these may be how the experience of adversity influences feelings of self-worth, mastery and the ability to develop positive and trusting relationships. In turn this may influence coping capacities and the ability to ask for help in a crisis (Gunnell *et al.* 2004). Mastery has been linked to lower levels of suicidal ideation (Fairweather *et al.* 2007). Childhood adversity and abuse have been linked to coping methods for stressors in adulthood linked to disengagement (problem avoidance, social withdrawal and self-criticism), although, surprisingly in this study, not to less social support (Leitenberg *et al.* 2004). This pattern of behavior may result from stress sensitization following childhood adversity which may hinder emotional processing of adult adverse events. Moreover, difficulties in dealing with adult adversity may accumulate in terms of perceived burden and severe interpersonal difficulties perpetuating and increasing suicidal ideas (Heikkinen *et al.* 1993).

A common theme across studies of risk factors for suicidal ideation and suicide is low social support and social isolation (Heikkinen *et al.* 1993; Johnson *et al.* 2002; Gunnell *et al.* 2004; Dennis *et al.* 2007). Our measures of social support were limited across the lifecourse and did not fully capture this dimension. Interpersonal skills deficits as one of the features of cluster B personality disorders with emotional dysregulation, disinhibition and ‘thwarted belonginess’ may be reasons for increased suicidal attempts in this patient group (May *et al.* 2012). Future lifecourse studies should attempt to capture the ways people cope with stressors, develop interpersonal relationships and ask for help in relation to suicidal ideation.

It is a strength that we had prospective measurements of childhood adversities and mediating factors at different life stages. To our knowledge this is the first study to examine the role of mediators of prospectively measured childhood adversity and its association with midlife suicidal ideation. It is a limitation that childhood sexual and physical abuse were reported retrospectively (Colman *et al.* 2016). Also our adversities were measured long before standard scales like the Parental Bonding Instrument had been developed and they were not designed to capture current conceptualizations of adversity. The main limitation of longitudinal cohort study analysis is sample attrition and missing data but we anticipate that multiple imputation has addressed this. Also, at present it is only possible computationally to perform relatively simple mediation analyses.

## Conclusions

Suicidal ideation and completed suicide are not equivalent, and less than 1 in 200 of those with suicidal ideation proceed to suicide (Gunnell *et al.* 2004). However, in the National Comorbidity Study the conditional probability of making a suicidal attempt among those with suicidal ideation was 57.9% with a suicide plan and 25.2% without a plan (Kessler *et al.* 1999) so that suicidal ideation should be considered an indicator of clinically significant risk both of suicidal attempt and major depressive disorder (Kessler *et al.* 1999).

Some elements of childhood adversity have an impact on midlife suicidal ideation after adjustment for mediating factors. This is in keeping with effects of adversity in critical periods in childhood having long-term consequences for mental health that may reflect effects on the developing brain and neuroendocrine responses to stress.

Understanding the pathways from adversity to suicidal ideation can inform suicide prevention (Gunnell *et al.* 2004). Interventions focused on parenting may be most effective in preventing childhood affective disorders and hence the transmission to adulthood (Shonkoff & Fisher, 2013). Additionally, interventions establishing mentors or significant others as alternative points of support and attachment during childhood would be a viable intervention pathway. Our results indicate that there are both childhood and adulthood factors that increase the risk of suicidal ideation and that interventions with internalizing, externalizing disorders and preventing relationship breakdown in adulthood may be relevant in prevention of the long-term consequences of childhood adversity.

## Supplementary material

For supplementary material accompanying this paper visit http://dx.doi.org/10.1017/S0033291716002336. click here to view supplementary material
