# Supplementary material for: Childhood adversity and midlife suicidal ideation
Source: Psychol Med. 2016 Oct 20;47(2):327–40. doi: 10.1017/S0033291716002336 (PMC5216460; doi:10.1017/S0033291716002336)
Supplement: Supplementary file 1 [file S0033291716002336sup001.docx]

**Table 6: Adjusted odds ratios (AOR) and 95% confidence intervals with supporting mediation effect showing the increase in odds for suicidal ideation and depressive ideas at 45y, for each childhood adversity**

| **Adversity** | **In the past week have you felt that life isn’t worth living** | | | | **Depressive Ideas** | | | |
| --- | --- | --- | --- | --- | --- | --- | --- | --- |
|  | AOR | 95%CI | Indirect % of Total Mediation Effect | | Indirect % of Total Mediation Effect | | | |
|  |  |  | **Dichotomised adversity scale** | | AOR | 95%CI | **Dichotomised adversity scale** | |
|  |  |  | Mean % | 95%CI |  |  | Mean % | 95%CI |
|  |  |  |  |  |  |  |  |  |
| Illness in the household | 2.15*** | 1.53,3.03 | 3.28 | 2.26, 5.41 | 1.32* | 1.06, 1.64 | 7.92 | 4.80, 21.23 |
| Neglected/underfed appearance (7-11y) | 1.48 | 0.80, 2.73 |  |  | 1.35 | 0.91, 2.00 |  |  |
| Maternal absence | 0.92 | 0.48, 1.74 |  |  | 0.96 | 0.68, 1.36 |  |  |
| Paternal absence | 2.03*** | 1.43, 2.90 | 3.50 | 2.17, 7.04 | 1.28* | 1.02, 1.59 | 8.67 | -0.35, 38.36 |
| In care | 1.94* | 1.11, 3.37 | 7.88 | -0.46, 29.54 | 1.41 | 0.97, 2.05 |  |  |
| Divorce of parents by age 16^1^ | 1.70** | 1.16, 2.50 | 8.17 | 3.9, 35.34 | 1.23 | 0.96, 1.57 |  |  |
| Parental physical abuse^2^ | 2.61*** | 1.74, 3.90 | 8.55 | 4.88, 18.61 | 2.40*** | 1.88, 3.06 | 8.10 | 5.83, 12.20 |
| Parental sexual abuse^2^ | 3.08*** | 1.68, 5.64 | 8.63 | 3.85, 31.29 | 2.66*** | 1.77, 3.99 | 7.86 | 4.87, 15.28 |
| Cumulative adversity scale        1 | 1.75* | 1.08, 2.84 |  |  | 1.36* | 1.05, 1.76 |  |  |
| 2 | 2.39*** | 1.42, 4.02 |  |  | 1.44* | 1.08, 1.92 |  |  |
| 3 or more | 3.79*** | 2.33, 6.15 |  |  | 1.94*** | 1.47, 2.56 |  |  |
| *p≤0.05, **p≤0.01, ***p≤0.001, ^1^ reported at 33y and ^2^ reported at 45y. | | | | | | | | |

**Adjusted for gender, social class at 42y, qualifications at 33y, life events at 45y, number of partnerships in adulthood, number of periods of unemployment in adulthood, emotional support at 42y, problem drinking at 33y or 42y, and long standing illness at 42y**
